# Tea regimen, a comprehensive assessment of antioxidant and antitumor activities of tea extract produced by Tie Guanyin hybridization†

**DOI:** 10.1039/c8ra00151k

**Published:** 2018-03-21

**Authors:** Xiaobin Zhang, Chengli Dai, Yuanyuan You, Lizhen He, Tianfeng Chen

**Affiliations:** The First Affiliated Hospital, Department of Chemistry, Jinan University Guangzhou 510632 China hlz6371@jnu.edu.cn tchentf@jnu.edu.cn

## Abstract

A comprehensive assessment was conducted in this study to examine the antioxidant and antitumor activities of tea extract produced by Tie Guanyin hybridization. Two radical-scavenging systems of assay *in vitro*, namely ABTS and DPPH assays, were used to investigate the antioxidant activity of the summer tea and autumn tea extract (STE and ATE) derived from the Jin Guanyin. The results indicated that the major active ingredients were catechins, and the theaflavin is rare in the STE and ATE. Moreover, STE and ATE could significantly suppress the growth of human breast cancer cells MDA-MB-231 in a dose-dependent manner, and wrecked the morphology of mitochondria, activated caspase families, leading to the cancer cell death by both apoptosis and cell cycle arrest pathways. Based on the results from an MDA-MB-231 xenograft nude mice model, STE could effectively prevent the tumor formation, and greatly improve the mice immunity and thus improve their living conditions. Taken together, ATE and STE could act as a healthy and prospective substitute for natural antioxidants and a promising prophylactic agent against cancer. This finding provides a great promising nutritional approach to treat diseases related with oxidative stress.

## Abstract 2

Herein we demonstrate that Jin Guanyin extracts shows antioxidative activity, thus inhibiting ROS generation, promoting mitochondrial fragmentations and caspase activations in cancer cells, finally leading cell apoptosis and cycle arrest.

## Graphical abstract

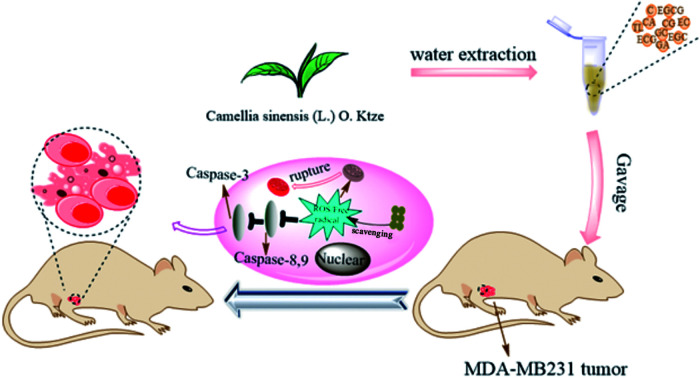

## Introduction

As is known to all, tea is the second most popular beverage all over the world,^1^ which is slightly inferior to water. Tea has beneficial properties such as anti-oxidative and anti-tumor capabilities and the effect of improving cardiovascular function.^2^ Oolong tea (semi-fermented), mainly produced in Fujian, Guangdong and Taiwan in China, is one of the groups that is classified from the tea based on the manufacturing process, the other two groups are green tea (non-fermented) and black tea (fully-fermented).^3,4^ Numerous studies have reported that drinking tea imparts various physiological and pharmacological benefits which include, anti-diabetic, anti-inflammatory, antioxidant, anticholesterolemic, anti-mutagenic and anti-carcinogenic activities.^5–10^ Jin Guanyin, also known as the no. 1 of Mingke tea (a new variety of tea that cultivated by the Tea Research Institute of Fujian Academy of Agricultural Sciences), is a new breed of clones that adopted the hybrid breeding method with the Tie Guanyin as the female parent and the golden GUI as the male from 1978 to 1999. Jin Guanyin, in accordance with the unique traditional oolong tea making process, which belongs to a semi-fermented tea. The quality characteristic of Jin Guanyin, “its shape is heavy as iron, the beauty is like Guan Yin” enjoys great fame both at home and abroad. The dry fine powder of the summer tea (STE) and autumn tea (ATE) that picked in different seasons is the main products of the Jin Guanyin tea. The active components playing key roles in most of the biological activities in tea are considered as catechins (also known as polyphenols).^11,12^ Moreover, tea polyphenols are well-known for possessing antioxidant capability. Both green tea and black tea are capable of working as potential antioxidants thanks to the presence of catechins and their ramifications in different forms.^13^ Tea contains as much as 30% soluble ingredients, which differ from the cultivar, the climate, the production region, and the processing and manual operations. The main constituents of tea are polyphenols, taking up 20–35% of tea's dry weight. Among which, catechins are the predominant group, counting for 60–80% of tea polyphenols. Four major substances including epigallocatechin-3-*O*-gallate (EGCG, consisting of 9–14% in tea's dry weight), epigallocatechin (EGC, 4–7%), epicatechin-3-*O*-gallate (ECG, 2–4%) and epicatechin (EC, 1–3%) provide 90% of the total catechin fraction in tea.^14,15^ It is well-known that tea polyphenols possess excellent antioxidative properties. Moreover, stronger antioxidative activity of green tea is mainly attributed to catechin derivatives such as epicatechin gallate and epigallocatechin gallate.^8,16–18^ Numerous studies have indicated that tea catechins and polyphenols are effective oxygen scavengers of free radicals/reactive oxygen species generated due to various oxidative stress.^19,20^ As the oolong tea is semi-fermented, the contents of the tea catechins and polyphenols are abundant in the extract of the tea. Hence, the STE and ATE are supposed to contain lots of tea catechins and polyphenols, as they are derived from the oolong tea.

Additionally, the health-promoting effect of tea has been known to us for a long time. The evidence above all support for that tea is effective for cardiovascular diseases and cancers to a certain extent and it is mainly thanks to the consist of catechins (flavanols), which possess particular antioxidative effects for human such as relieving vascular pressure, reducing triglyceride and cholesterol, as well as inhibiting the oxidation process of low-density lipoprotein.^14,15,21–23^ The anti-cancer effect is closely related to the induction of cell cycle arrest, apoptotic cell death pathways in cancer cells and some other anti-cancer mechanisms.

Taking into account that tea drinks are widely used as a health care product for its and especially the lack of experimental research on the antitumor activity of STE and ATE (belong to oolong tea), the further investigation of the STE and ATE about free radical scavenging and anti-cancer mechanism was carried to evaluate the antioxidative activity take advantage of ABTS˙^+^ and DPPH˙ free radicals, and capability of anticancer activity on MDA-MB-231 (human breast cancer cells) and SW480 (human colon cancer cells) cancer cell lines. We clarified the mechanisms accounting for the anti-cancer actions of the STE and ATE. In order to further testify whether STE can inhibit malignant formation and growth of tumors, a nude mice model of MDA-MB-231 transplantation xenograft model was constructed in our study.

**Fig. 1:**
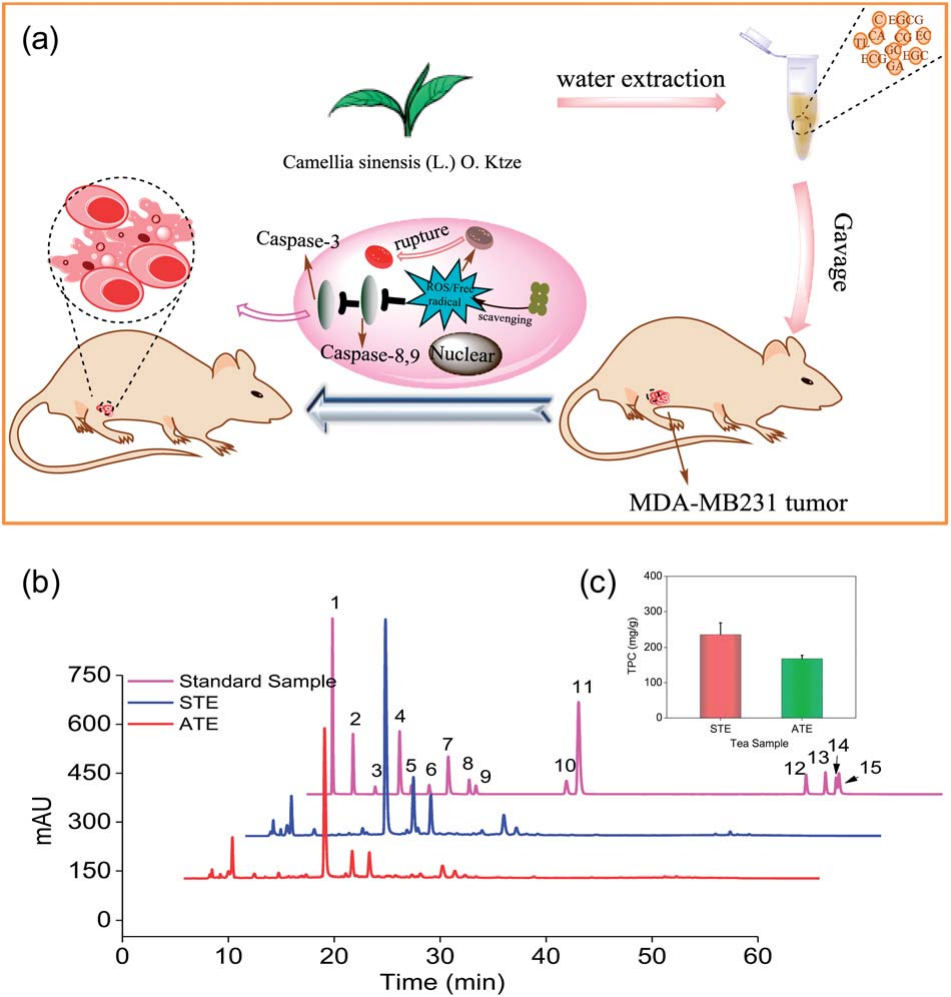
Anticancer mechanism of STE and HPLC analysis of the active ingredients in different Jin Guanyin extracts. (a) The anticancer mechanism of STE. (b) Representative HPLC-UV chromatography acquired at 278 nm of 15 standards samples, STE and ATE, respectively. (c) The contents of total phenolic contents (TPC) (as gallic acid equivalents) in ATE and STE (1: A; 2: GA; 3: GC; 4: TL; 5: EGC; 6: C; 7: CA; 8: EC; 9: EGCG; 10: ECG; 11: CG; 12: TF1; 13: TF2; 14: TF3; 15: TF4).

Based on the preliminary screening, the MDA-MB-231 breast cancer cell line was selected as the cell model to evaluate the *in vitro* and *in vivo* antitumor effects of STE and ATE (Fig. 1a). Antioxidative activities, caspase activations, inhibiting ROS generation, mitochondrial fragmentation, and MDA-MB-231 xenograft nude model were conducted in this study. The results showed that STE and ATE had the excellent protective effect to resist against MDA-MB-231 cancer cells both *in vitro* and *in vivo*.

## Experimental section

### Materials and reagents

The reference standard of caffeine, EC, ECG, CG, C, CA, EGC, and EGCG (≥98%) and those theaflavins (TF1, TF2, TF3 and TF4) (≥90%) was purchased from Chen du purify Co. (Cheng du, China), Folin–Ciocalteu's phenol reagent were purchased from Sigma (St. Louis, MO), Methanol (HPLC grade), 85% phosphoric acid (HPLC grade), acetonitrile (HPLC grade) and Milli-Q water were filtered through a 0.45 μm membrane before we use. 6-Hydroxy-2,5,7,8-tetramethylchromane-2-carboxylic acid (trolox), 2,2′-azinobis-3-ethylbenzothiazolin-6-sulfonic acid (ABTS˙^+^), 1,1-diphenyl-2-picryhydrazyl (DPPH˙), propidium iodide (PI), thiazolyl blue tetrazolium bromide (MTT), 4′,6-diamidino-2-phenylindole (DAPI), bicinchoninic acid (BCA), sodium selenite and all other chemicals were bought from Sigma-Aldrich (St. Louis, MO, USA). Fetal bovine serum (FBS) and the antibiotic mixture (penicillin–streptomycin) were purchased from Invitrogen (Carlsbad, CA, USA). Caspase-3/8/9 was purchased from Cell Signaling Technology (Beverly, MA, USA). Caspase-3/8/9 substrates were obtained from Biomol (Germany).

### Preparation of tea extract

The dry fine powder of summer and autumn tea of Jin Guanyin that added 1/2 (w/w) and 1/1 (w/w) tea additive respectively were collected from the local tea processing plant as granular powder of a particular local brand. The tea additive includes 19.83% coniferous cherry powder, 54% sorbitol, 25.3% d-mannose and 0.87% stearate magnesium stearate. Dry fine powder was stored at 4 °C and when necessary it was dissolved in phosphate buffered saline (PBS) to make 5 mg mL^−1^ working solution.

### Determination of total polyphenol content (TPC) of STE and ATE

TPC was determined by spectrophotometry against gallic acid (GA) as standard by the Folin–Ciocalteu method.^24^ Absorbance of these solution was measured at 765 nm against water. Each sample was measured in triplicate. The same procedures were repeated for standard solution. The TPC was expressed as gallic acid equivalents (GAE) in mg of GA/g of tea extract.

### Determination of catechins and theaflavins content of STE and ATE

High performance liquid chromatography (HPLC)^25^ was used to analyse the content of catechins and theaflavins in STE and ATE. HPLC was performed using a 1260 infinity ii chromatography system purchased from Agilent Technologies. STE and ATE were injected onto a SiO_2_ column (250 × 4.6 mm), previously equilibrated with a solution composed of solvent A (acetonitrile) and solvent B (0.4% aqueous phosphoric acid, v/v). Compounds were eluted from the column according to the following program: 7–15% A in 0–13 min, 15–20% A in 13–35 min, 20–50% A in 35–70 min, 50–80% A in 70–90 min, 80–7% A in 90–115 min. The flow-rate was 1.0 mL min^−1^ and the effluent were monitored at 278 nm for acquiring chromatograms.

### ABTS˙^+^ free radical scavenging assay

The antioxidative activities of STE and ATE were measured by ABTS˙^+^ free radical scavenging assay as previously described.^26,27^ Briefly, 0.1 mL ABTS reagent was mixed with 0.1 mL of sample or PBS buffer and trolox. The absorbance was measured at 734 nm and half maximal inhibitory concentration (IC_50_) was calculated to evaluate antioxidant capacity of the STE and ATE. The scavenging assay was performed on the spectrophotometer (Spectra Max M5, MD, USA).

### DPPH˙ free radical scavenging assay

The DPPH˙ free radical scavenging activity of STE and ATE was determined using a spectrophotometer (Spectra Max M5, MD, USA) according to the previous method.^26^ Briefly, test samples and DPPH˙ radical solution was mixed at the ratio of 1 : 1 (the final concentration of DPPH˙ solution was 2.0 × 10^−4^ M), and then they were shaken up. The decrease in absorbance caused by the addition of the tea sample was compared with a standard curve that made by trolox. The change in absorbance of the mixture that left to stand for 5 min at 515 nm was measured. The antioxidant capacity of the STE and ATE were evaluated by calculating half maximal inhibitory concentration (IC_50_) value.

### Cell culture and MTT assay

All the human cell lines that involved in this experiment, including Hela (human cervical cancer cells), HepG2 (hepatocellular carcinoma cells), SW480 (colon cancer cells), MDA-MB-231 (breast cancer cells) and normal cells like WI38 (human lung cells), L02 (human liver cells), were purchased from American Type Culture Collection (ATCC®, Manassas, Virginia). These cell lines were incubated in DMEM media supplemented with penicillin (100 units per mL), streptomycin (50 units per mL) and fetal bovine serum (10%) at 37 °C in a humidified incubator with 5% CO_2_ atmospheres. Reagents for cell cultures were purchased from GIBCO. Cell viability induced by the STE and ATE was determined by MTT assay, based on previous study.^28^ The colour intensity of the formazan solution that reflects the growth state of the cells was measured at 570 nm using a micro-plate spectrophotometer (Spectro Amax™ 250).^29^

### Cell cycle analysis

The cell cycle distribution was analysed by flow cytometry as previously described.^30^ In short, MDA-MB-231 cells treated with STE and ATE respectively were washed with PBS and then treated with 5% trypsin, then fixed in 75% ethanol overnight at −20 °C. Subsequently, the fixed cells were stained with PI in darkness. The stained cells were analysed by using FC-500 flow cytometer (Beckman Coulter, Miami, FL). Multicycle software (Phoenix Flow Systems, San Diego, CA) was used to analyse the cell cycle distribution. DNA histogram was represented by the proportion of cells in G0/G1, S and G2/M phases. Apoptotic cells with hypodiploid DNA content were measured by quantifying the sub-G1 peak in the cell cycle pattern. 10 000 events were recorded in each experiment group.

### Caspase activity assay

Caspase activity in MDA-MB-231 cellular proteins were evaluated by using the caspase activity kit (BD Biosciences) as reported before.^31^ In particular, the MDA-MB-231 cells were split by cell lysis buffer and the protein concentrations were measured by used of BCA assay. Subsequently, the cellular protein solution and specific caspase substrates (Ac-DEVD-AMC for caspase-3, Ac-IETD-AMC for caspase-8, and Ac-LEHD-AMC for caspase-9) were mixed then the mixtures were incubated at 37 °C for 2 h. Caspase activity of MDA-MB231 cells induced by STE and ATE was determined by fluorescence microplate reader (ELX800, Bio-Tek, American).

### Measurement of intracellular ROS generation

The relative levels of ROS were determined by a fluorometric assay (DHE assay).^32,33^ The generation of ROS was determined by fluorescence intensity using the Multi-function fluorescent enzyme meter (Bio-Tek, ELX 800, American) with the excitation and emission wavelengths set at 300 nm and 600 nm, respectively. Relative DHE fluorescence intensity of treated cells was expressed as a percentage of control (as 100%).

### Mitochondrial fragmentation analysis

Mitochondrial fragmentation analysis was carried out as previously reported.^29^ In short, mitochondria and nucleus of the MDA-MB-231 cells were stained with Mito Tracker Red CMX Ros and DAPI, respectively. After that, the cells were treated with STE and ATE respectively for 12 h, then the cells were photographed under a monochromatic Cool SNAPFX camera (Roper Scientific, USA).

### Evaluation of antitumor activity *in vivo*

In order to further study the anti-cancer effect of STE *in vivo*, we constructed the MDA-MB-231 *in vivo* xenograft model, the specific experimental methods were described as previously reported.^34,35^ Briefly, the *in vivo* antitumor activity of STE was measured after 24 days' daily irrigation of the nude mice. The blood sample (72 h) of the nude mice was subjected to haematological analysis at Guangzhou Overseas Chinese Hospital. All animal procedures were performed in accordance with the Guidelines for Care and Use of Laboratory Animals of Jinan University and approved by the Animal Ethics Committee of Jinan University.

### Statistical analysis

All results were expressed as mean ± SD, which were carried out in three independent experiments results. One-way analysis of variance method was used in multiple group comparisons, and the statistical software package (SPSS Inc., Chicago, IL) was used for statistical analysis. Statistical significance between multiple groups was analysed at *P* < 0.05 (*) or *P* < 0.01 (**) levels.

## Results and discussion

### Measurement of the active ingredients in STE and ATE

As shown in Fig. 1b, the typical HPLC-UV chromatogram at 278 nm of the STE, ATE and 15 standard samples. The retention times and absorption spectra were compared with the commercially available catechins and theaflavins. The identification of the peaks and their retention times of the STE and ATE were compared to the standard compounds, subsequently the contents of 15 kinds of the active ingredients in the STE and ATE were calculated (Table 1). The result demonstrated that the contents of GC, EGCG and CA are at the same level in STE and ATE, and there was significant difference of the contents of some ingredients such as GA, EGC, EC and ECG between STE and ATE.

For example, the contents of ECG in STE were 3.1%, which was 3 times as that of in ATE. In addition, it was obvious that the contents of TF3 and TF4 in ATE was below the minimum detection limit. Additionally, as shown in (Fig. 1c), the TPC contents of STE (236.0 mg g^−1^), which was much higher than that of in ATE (168.0 mg g^−1^). Taken together, it was manifested that EGC, EC, ECG, EGCG and GC was the predominant components of catechins in STE, while GC and EGCG was the major components of catechins in ATE, it was apparent that the theaflavins was rare both in STE and ATE.

**Table 1:** Chemical composition analysis of STE and ATE by HPLC-UV analysis

Peak compound	Time (min)	Retention	Contents (%)	
			STE	ATE
1	2.436	A	0.63	0.12
2	4.375	GA	0.087	1.4
3	6.467	GC	2.1	2.7
4	8.771	TL	0.044	0.15
5	9.890	EGC	3.0	0.59
6	11.56	C	0.97	0.28
7	13.36	CA	4.3	3.6
8	15.34	EC	2.0	0.49
9	15.97	EGCG	2.1	2.7
10	24.52	ECG	3.1	0.92
11	25.66	CG	0.36	0.51
12	47.14	TF1	0.054	0.052
13	48.97	TF2	0.098	0.051
14	49.98	TF3	0.088	0
15	50.24	TF4	0.15	0

### Determination of the optimum wavelength

**Fig. 2:**
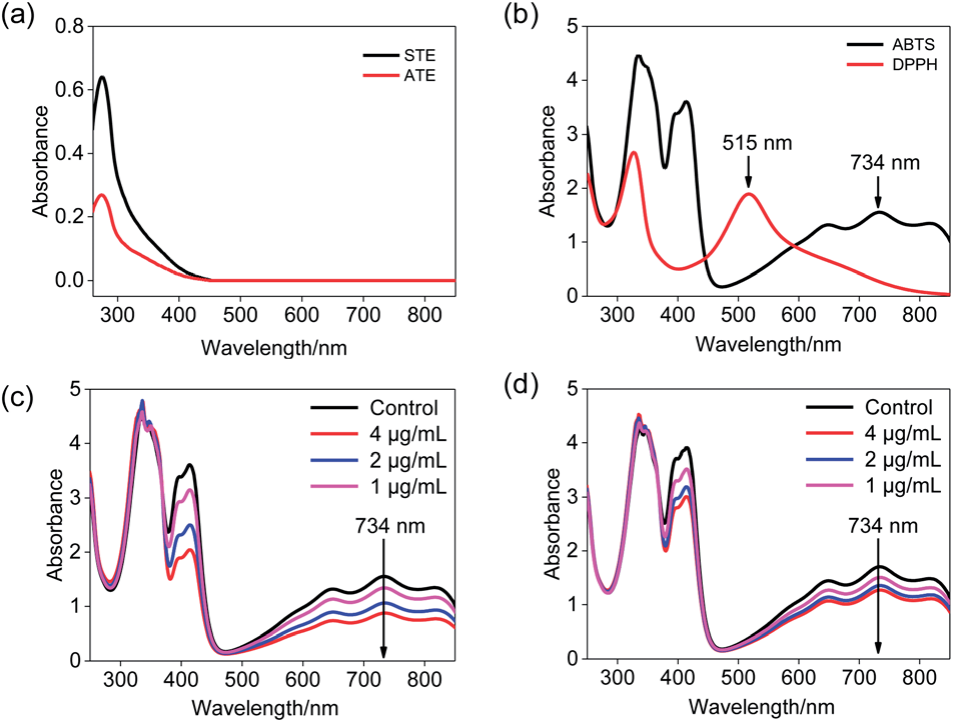
Ultraviolet absorbance spectra of Jin Guanyin extracts. (a) Absorbance spectra of STE and ATE. (b) Absorbance spectra of the ABTS˙^+^ and DPPH˙ solutions. (c) Changes of absorbance spectra in ABTS˙^+^ solution with the addition of STE. (d) Changes in absorbance spectra of ABTS˙^+^ solution with addition of ATE.

ABTS˙^+^ free radical scavenging has been widely used in the determination of total antioxidative capacity of the biological samples.^29^ In this study, the ABTS solution was subjected to UV-Vis spectral scanning. As shown in Fig. 2b, ABTS˙^+^ work solution showed characteristic absorption peaks at 734 nm and 415 nm, and the ATE and STE had certain ultraviolet absorption at a range of 300–420 nm (Fig. 2a). Additionally, the absorption peak at 734 nm of ABTS descended in a dose-dependent manner and relatively good linearity correlation after the addition of STE and ATE (Fig. 2c and d). The results above indicated that the change in absorption peak at 734 nm can reflect the sample scavenge ABTS˙^+^ free radicals to a certain degree, so the 734 nm was ascertained as the detection wavelength of ABTS˙^+^ free radical scavenging experiments. As for the selection of detection wavelength in DPPH˙ free radical scavenging experiments (Fig. 2a and b), DPPH˙ solution showed characteristic absorption peak at 515 nm, while the ATE and STE had no ultraviolet absorption at the wavelength above 450 nm, thus the optimum detection wavelength of DPPH˙ free radical scavenging experiments was 515 nm.

### Reaction kinetics of ABTS˙^+^ and DPPH˙ systems

In the ABTS˙^+^ and DPPH˙ radical scavenging experiments, the determination of the response time of the system was a key factor for the different antioxidants to be tested.^36^ In order to understand the reaction kinetic characteristics of ABTS and DPPH systems, the absorbance change pattern of ABTS˙^+^ and DPPH˙ radical solutions was measured after adding STE, ATE and trolox, respectively.

**Fig. 3:**
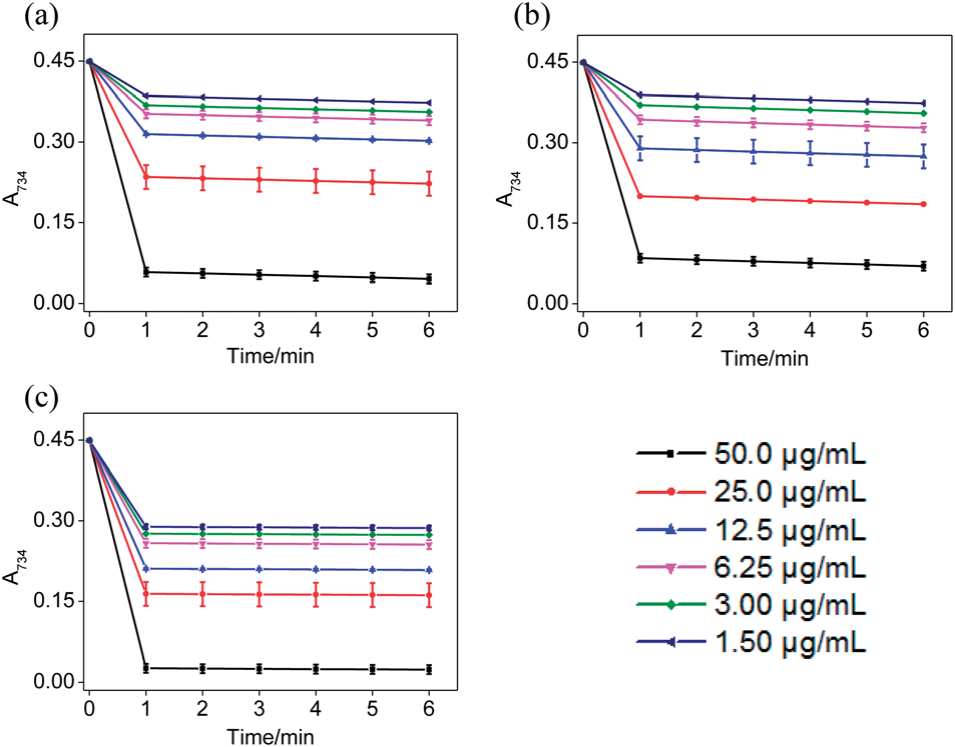
The absorbance inhibition level of ABTS˙^+^ (A_734_) after addition of Jin Guanyin extracts. (a) Changes in absorbance of ABTS˙^+^ solution with the addition of STE. (b) Changes in absorbance of ABTS˙^+^ solution with the addition of ATE. (c) Changes in absorbance of ABTS˙^+^ solution with the addition of trolox. Each value represents means ± SD (*n* = 3).

**Fig. 4:**
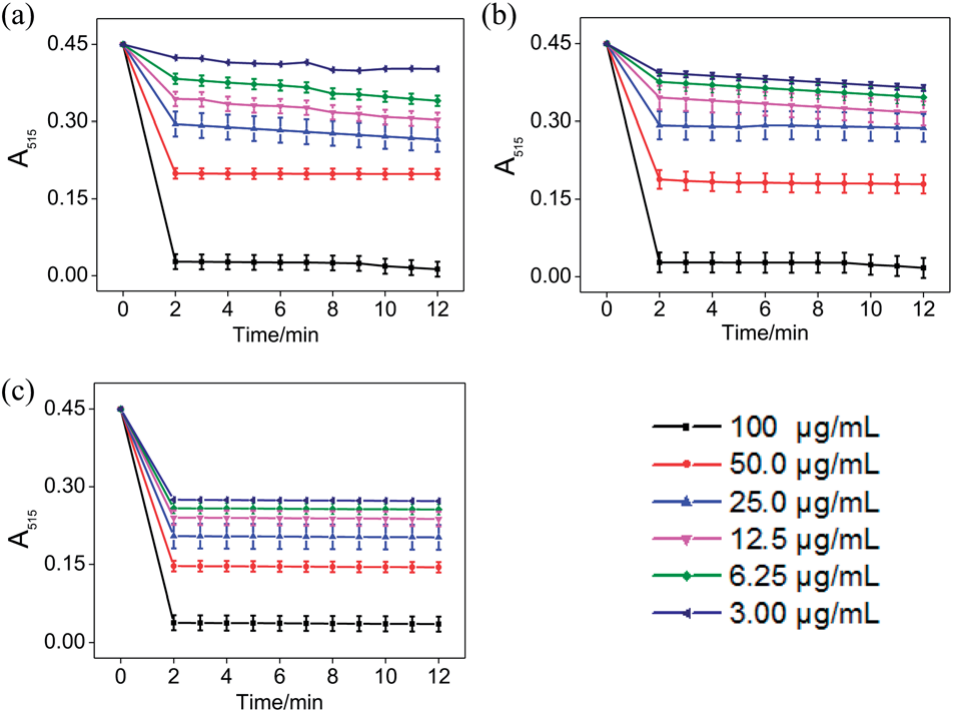
The absorbance inhibition level of DPPH˙ (A_515_) after addition of Jin Guanyin extracts. (a) Changes in absorbance of DPPH˙ solution with the addition of STE. (b) Changes in absorbance of DPPH˙ solution with the addition of ATE. (c) Changes in absorbance of DPPH˙ solution with the addition of trolox (standard oxidant). Each value represents means ± SD (*n* = 3).

According to Fig. 3, when different concentrations of STE, ATE and trolox were added to the ABTS˙^+^ system respectively, the characteristic absorption peaks (A_734_) of the ABTS system decreased significantly in 60 seconds, and then reached to a steady state after 6 minutes. The results indicated that 6 minutes was the optimal time point for further study of dose-dependent effects for STE and ATE in the ABTS˙^+^ system. Similarly, it was manifested that 120 s was the excellent time point for further study of dosage effects for STE, ATE and trolox in the DPPH˙ system due to the characteristic absorption peaks (A_515_) of the DPPH˙ system decreased significantly in 120 s (Fig. 4), and there was a slight fluctuation in 12 minutes, and finally tended to be steady after 30 minutes (Fig. S1†).

### Antioxidative activity in cell-free systems

**Fig. 5:**
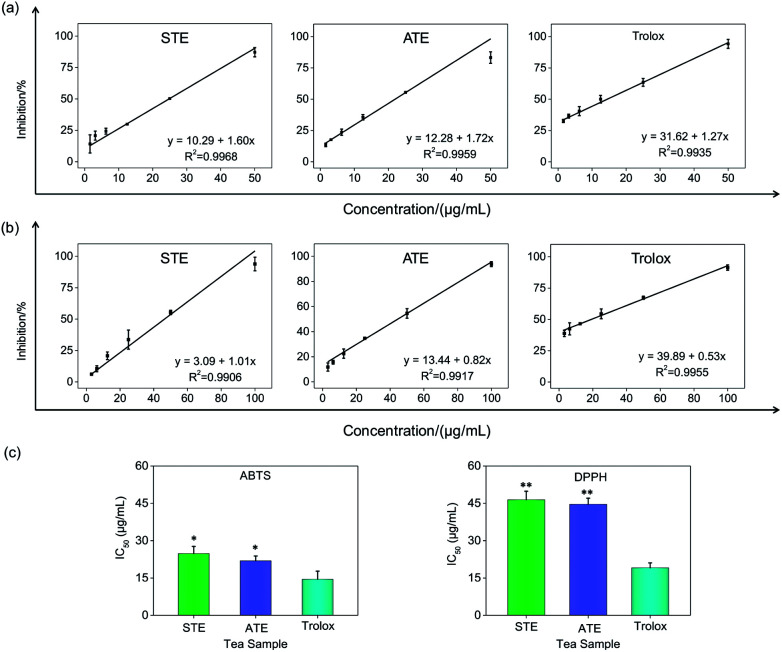
Antioxidative activities of the Jin Guanyin extracts by ABTS and DPPH assays. (a) Dose-dependent inhibition actions of STE and ATE and trolox on ABTS˙^+^ and its linearity correlation. (b) Dose-dependent inhibition actions of STE and ATE and trolox on DPPH˙ free radicals and its linearity correlation. (c) IC_50_ values of STE. Each value represents means ± SD (*n* = 3). * and ** indicate statistical difference at *P* < 0.05 and *P* < 0.01 level by comparing with trolox group.

On account of the ABTS and DPPH radical scavenging test could accommodate lots of samples in a short period and were sensitive enough to detect natural compounds' antioxidative activity at low concentrations, it was utilized in the following study for a primary screening of the STE and ATE free radical scavenging activity. The IC_50_ values of the corresponding samples (STE, ATE and trolox) were calculated based on the scavenging levels of ABTS˙^+^ and DPPH˙ radicals under the selected wavelengths and reaction times, the oxidative activity was evaluated refer to the IC_50_ value. For ABTS˙^+^ system, in the concentration range from 1.5 to 50 μg mL^−1^, all three samples showed good linear relationships to free radical scavenging (Fig. 5a), thereby the IC_50_ values could be calculated by the ABTS˙^+^ free radical scavenging experiment. In detail, the IC_50_ values of STE, ATE and trolox were 24.82 ± 2.9, 21.93 ± 1.9 and 14.47 ± 3.3 μg mL^−1^ (Fig. 5c), respectively, which suggested that the STE and ATE showed excellent total antioxidant activity to a great extent. Similarly, for the DPPH˙ system, STE and ATE and trolox (as positive control) showed good linear relationship to free radical scavenging in the range from 3.00 to 100 μg mL^−1^ (Fig. 5b). As shown in Fig. 5c, the IC_50_ values of STE, ATE and trolox were 46.45 ± 3.4, 44.59 ± 2.5 and 19.08 ± 2.0 μg mL^−1^, respectively, which indicated that STE and ATE also exhibited an excellent scavenging effect on lipophilic free radical (DPPH˙). Based on the results above, it could be concluded that both the STE and ATE demonstrated good resistance oxidative activity from the results of ABTS˙^+^ and DPPH˙ free radical scavenging assays, and the inhibition level of water-soluble free radicals (ABTS˙^+^) was significantly higher than that of the lipo-soluble free radical (DPPH˙). And that was coincident with the results above.

### Cytotoxicity effects of STE and ATE

**Fig. 6:**
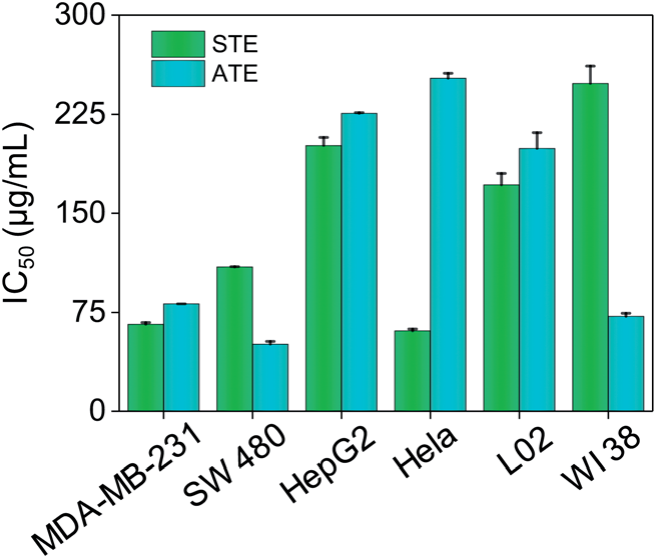
Cytotoxic effects of STE and ATE by MTT assay. Cytotoxicity effects of STE and ATE on different cancer and normal cells. Each value represents means ± SD (*n* = 3).

The cytotoxicity of STE and ATE toward cancer cell lines and normal cell lines were examined by MTT assay. HepG-2, Hela, MDA-MB-231, SW480 cancer cell lines and WI 38 (human lung cells), L02 (human liver cells) normal cell lines were chosen in this test. As shown in Fig. 6, the IC_50_ values of STE toward MDA-MB-231, SW480 and Hela cancer cells were 65.90 ± 1.4, 109.1 ± 0.22 and 60.90 ± 1.4 μg mL^−1^, respectively. While STE seemed no obvious effects toward HepG2 cancer cells (IC_50_ value was about 201.3 ± 6.1 μg mL^−1^). What's more, the IC_50_ value of STE toward normal cells seemed much higher compared with cancer cells as mentioned above (IC_50_ value was about 171.4 ± 8.7 μg mL^−1^ for L02 cells and 2448 ± 18 μg mL^−1^ for WI 38 cells). Besides, the IC_50_ values of ATE toward MDA-MB-231, SW480 and WI38 cells were 81.40 ± 0.037, 50.83 ± 2.1 and 71.95 ± 1.9 μg mL^−1^, respectively. While the IC_50_ values of ATE toward HepG2 and Hela cancer cells were 225.7 ± 0.62 and 252.0 ± 3.8 μg mL^−1^ (Table S1†), respectively, which suggest that HepG2 and Hela cancer cells are not sensitive to ATE. Also, Fig. 6 demonstrated that ATE exhibited cytotoxicity to WI 38 lung cells (IC_50_ value was about 71.95 ± 1.9 μg mL^−1^). In addition, four kinds of additives introduced into the tea extract did not show significant inhibiting effects to cancer cells (Fig. S2†). Additionally, the different sensitivity of STE and ATE toward the cancer cells might due to their diversified cellular protein expression profiles.^37^ Taken together, these results demonstrate that both STE and ATE showed obvious cytotoxicity to MDA-MB-231 and SW480 cancer cells, while HepG2 cancer cells seemed insensitive to STE or ATE. Consequently, MDA-MB-231 and SW480 cancer cells were selected to further illuminate the anticancer mechanism of STE and ATE in this work.

### Cancer cell apoptosis induced by STE and ATE

**Fig. 7:**
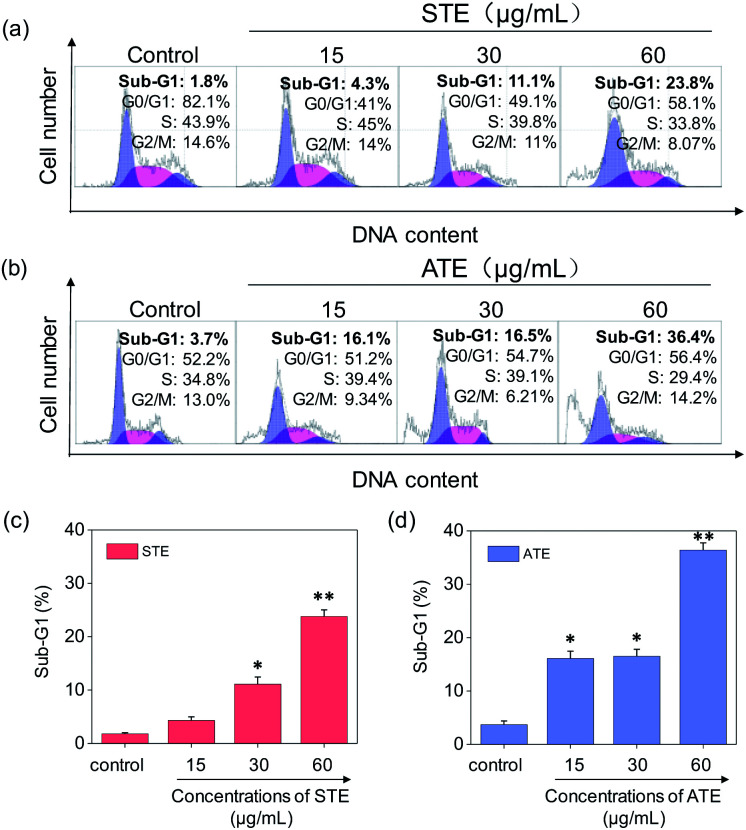
Flow cytometric analysis of MDA-MB-231 cells with the treatment of Jin Guanyin extracts. (a) and (b) Cell cycle distribution of MDA-MB-231 cells after being treated with STE and ATE. (c) and (d) Quantitative analysis of sub-G1 proportion by STE or ATE in MDA-MB-231 cells. Each value represents means ± SD (*n* = 3). * and ** indicate statistical difference at *P* < 0.05 and *P* < 0.01 level by comparing with control group.

**Fig. 8:**
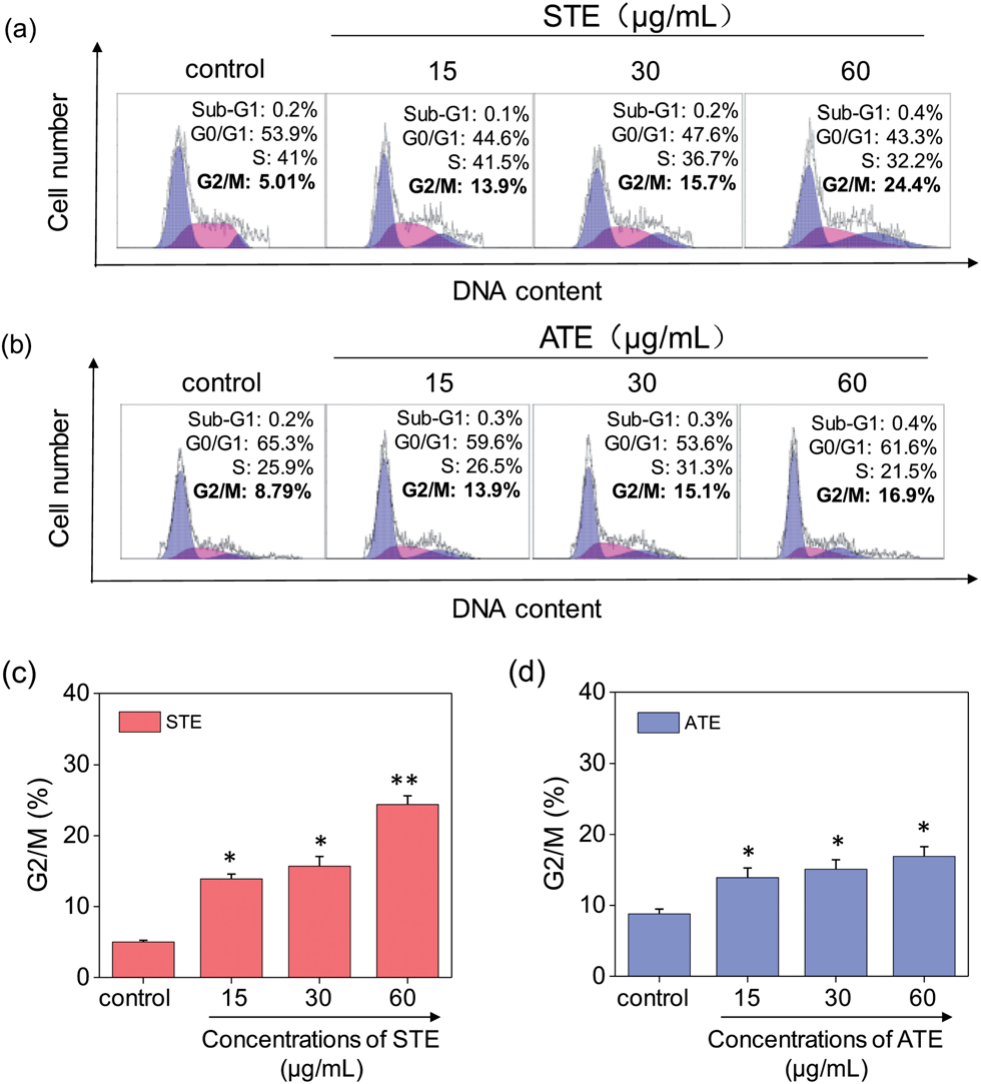
Flow cytometric analysis of SW480 cells with the treatment of Jin Guanyin extracts. (a) Cell cycle distribution of SW480 cells after the treatment of STE by PI-flow cytometric analysis. (b) Cell cycle distribution of SW480 cells after the treatment of ATE by PI-flow cytometric analysis. (c) Quantitative analysis of G2/M proportion induced by STE in SW480 cells. (d) Quantitative analysis of G2/M proportion induced by ATE in SW480 cells. Each value represents means ± SD (*n* = 3). * and ** indicate statistical difference at *P* < 0.05 and *P* < 0.01 level by comparing with control group.

Cell apoptosis and cycle arrest are considered as two major mechanisms to cause the cell death.^38–40^ Apoptosis plays an important part in different kinds of biological systems, including normal cell cycle, the immune system, embryonic development, morphologic change, and chemical-induced cell death.^41^ Flow cytometry was conducted to investigate the cell cycle distributions of MDA-MB-231 and SW480 affected by STE and ATE. According to Fig. 7a and b, the STE and ATE caused a significant cell death to MDA-MB-231 breast cancer cells after incubation for 72 h, as shown a significant ascendant sub-G1 peaks (Fig. 7c and d). Furthermore, both oolong tea extracts led cell death in a dose-dependent manner. For instance, the concentration of STE and ATE increased from 15 to 60 μg mL^−1^, sub-G1 peaks ascended from 4.3% to 23.8% (1.8% for control group), 16.1% to 36.4% (3.7% for control group), respectively. The results showed that STE and ATE could induce MDA-MB-231 cell death through cell apoptosis pathway. It was worth mentioning that when using the same treatment approach to SW480 cells (Fig. 8a and b), cycle arrest in G2/M phase was predominant in STE and ATE toward SW480 cells. As the sample concentrations changed from 15 to 60 μg mL^−1^, the G2/M peaks rise up from 13.9% to 24.4% (5.01% for STE control group), 13.9% to 16.9% (8.79% for ATE control group), respectively. While there were no significant changes in sub-G1 peak, which further elucidated the mechanism that STE and ATE inhibited the SW480 cells growth by arresting the cells in G2/M phase, slightly induced the cells apoptosis. The reasons accounting for the different response of different cell lines to STE and ATE could relate to the different protein expression profiles in the different cell types.^42,43^ For instance, under the same experimental condition, both STE and ATE could cause MDA-MB-231 and SW480 cancer cell death through activation of different pathways, such as apoptosis or cycle arrest (Fig. 7 and 8).

### Caspase activation induced by STE and ATE

**Fig. 9:**
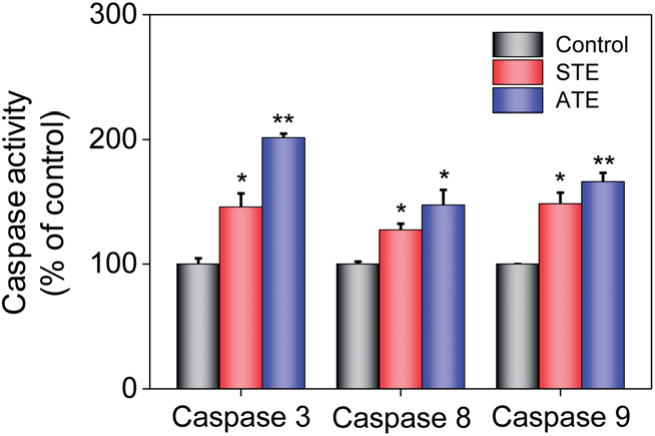
Quantitative analysis of caspase activation levels triggered by STE and ATE. Each value represents means ± SD (*n* = 3). * and ** indicate statistical difference at *P* < 0.05 and *P* < 0.01 level by comparing with control group.

Caspase family members are considered as a family group of cysteine proteases, and they play an important role in the cell apoptosis.^44,45^ Furthermore, caspase-3 plays a vital role in diseases induced by cell apoptosis, while caspase-8 and caspase-9 work as initiators of death receptor-mediated and mitochondria-mediated apoptotic pathway, respectively. Caspase activity assay was conducted to quantify the activation degrees of caspase-8, -9, and -3 in the MDA-MB-231 breast cancer cells after the treatment of STE and ATE. The results elucidated that STE and ATE suppressed the growth of cancer cells through triggering a remarkable activation of caspase-3 (145.84% for STE and 201.51% for ATE, 100% as control), caspase-8 (127.28% for STE and 147.41% for ATE, 100% as control), and caspase-9 (148.42% for STE and 166.13% for ATE, 100% as control) pathways in MDA-MB-231 cells (Fig. 9), which demonstrated that not only the death receptor-mediated pathway but also the mitochondria-mediated pathway was involved in the cancer cell apoptosis induced by STE and ATE. It could be supposed that the activation degree of caspase-9 had a markedly higher level compared with caspase-8 in MDA-MB-231 cells after the addition of STE and ATE. As a group, these results manifested that STE and ATE could inhibited MDA-MB-231 cell growth mainly through a mitochondria-mediated apoptotic pathway.

### Mitochondrial fragmentation induced by STE and ATE

**Fig. 10:**
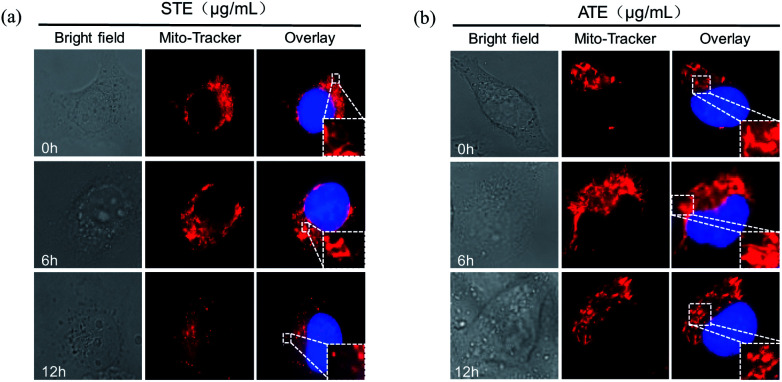
Morphological changes of mitochondria due to Jin Guanyin extracts. (a) The morphological change of mitochondria induced by STE in MDA-MB-231 cells. (b) The morphological change of mitochondria induced by ATE in MDA-MB-231 cells. Original magnification: 100×.

As is known to all, the mitochondria are the energy factories of cellular metabolic activities, but a variety of factors can result in damages to the structure and function of mitochondria, even further induce cell apoptosis.^46,47^ The results in Fig. 10a and b showed that healthy mitochondrial network (0 min) appeared filamentous, extending widely throughout the cytoplasm. The treatment of STE and ATE brought about obvious mitochondrial fragmentation, which displayed certain attack after 6 h of treatment, followed by progressive increase to 12 h. These evidences further confirmed the critical role of STE and ATE in the activation of mitochondrial-mediated apoptosis.

### Down-regulation of intracellular ROS generation induced by STE and ATE

**Fig. 11:**
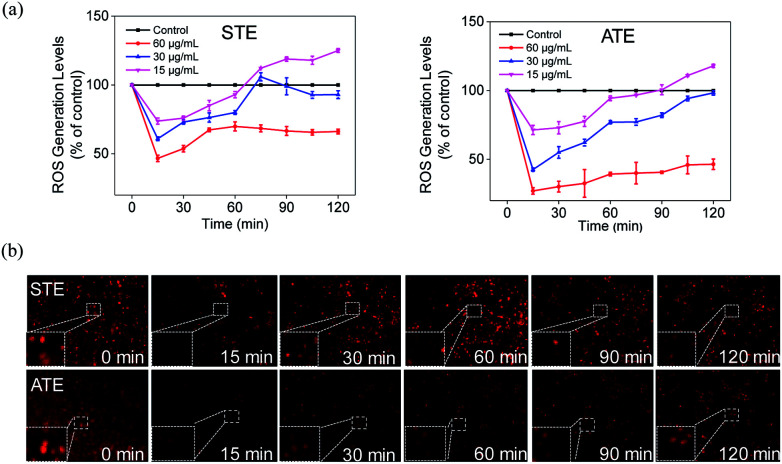
Decrease of intracellular ROS levels induced by Jin Guanyin extracts. (a) Intracellular ROS generation after treatment with STE and ATE, respectively. (b) Fluorescence images of ROS generation in MDA-MB-231 cells incubated with STE and ATE at indicated time points. Original magnification: 10×. Each value represents means ± SD (*n* = 3).

The imbalance of ROS and RNS has been considered as the principal action mechanism in the oxidative damage of islet cells.^48^ The excess amount of ROS are compelled to attack many components of DNA, thus causing DNA damage.^49^ Additionally, the appropriate level of ROS is seemed to be a crucial factor in all kinds of cell signal ways.^28,50–52^ In order to study whether STE and ATE could trigger ROS-mediated apoptosis pathway, the intracellular ROS level was monitored by detecting the fluorescence intensity of DHE. As shown in Fig. 11a, ROS generation level of MDA-MB-231 cells descended sharply after the exposure to STE and ATE with different concentrations in 15 min, after which picked up slowly within 120 minutes, and both STE and ATE inhibited ROS generation in a dose-dependent manner. Likewise, we observed STE down-regulate ROS level (60.92%, 30 μg mL^−1^), which was higher than ATE (42.53%, 30 μg mL^−1^) at 15 minutes in MDA-MB-231 cells. That is to say, STE and ATE could lead to the decrease of ROS at early stage (around 90 min) during the period of exposure. To vividly demonstrate the ROS level that STE and ATE could down-regulate, the fluorescence images of MDA-MB-231 cells were collected by a fluorescence microscope, Fig. 11b showed that the fluorescence intensity was the weakest in 15 min, and then got brighter in following period, the fluorescence intensity at other times was weaker than that at beginning, which was consistent with the prior result in Fig. 11a. Also, we found the same phenomena by DCFDA in Fig. S3,† the fluorescence intensity got the weakest in 15 min, and then appeared brighter subsequently. Taken together, the significant changes of ROS generation at early stage (DHE and DCFDA) in MDA-MB-231 cells could induced the cell apoptosis, which was mainly due to the involvement of STE and ATE.

### Acute toxicity evaluation

According to the observation on the xenograft nude mice, 7 days after the drug administration, the fed with STE (12 g kg^−1^) were dead suddenly, while the mice fed STE (6 g kg^−1^) were too excited and losing weight acutely, however the groups that treated with STE of 1.5 and 3 g kg^−1^ seemed to be in good growth status, where were no significant difference (*P* < 0.05) compared with the control group. Moreover, there was no observable abnormality in physical appearance in the two groups of mice during the experiment. It could come to a conclusion that an excessive dose of STE showed some toxic side effects to the mice. Thus, the administration dose (1.5 and 3 g kg^−1^) was selected for further *in vivo* antitumor evaluation.

### *In vivo* anti-tumor efficacy evaluation

**Fig. 12:**
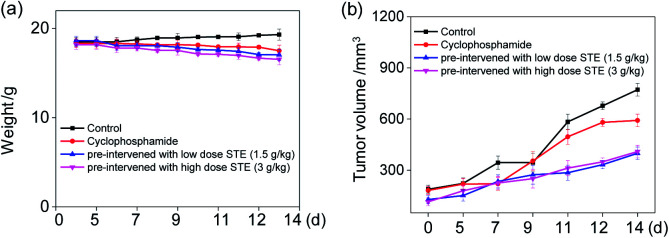
*In vivo* therapeutic effects of STE in MDA-MB-231-bearing nude mice. (a) The weight of nude mice through different treatments in 14 days. (b) Tumor volume index of different treatments in 14 days. Each value represents means ± SD (*n* = 3).

To further examine the anticancer potential of oolong tea extract *in vivo*, we assessed its therapeutic efficacy to the nude mice cultivated with MDA-MB-231 xenografts, and they were pre-treated through administration by gavage with STE (q.d.). As shown in Fig. 12b, after intragastric administrated with STE at different concentrations for 14 days, the tumor volume was significantly inhibited comparing with the control group. For instance, the tumor volume in pre-treatment with STE (3 g kg^−1^) declined at 14 days from 722 mm^3^ (control group) to 399 mm^3^. By the way, there was no significant difference in mice weight among each group (Fig. 12a). Futhermore, there was no significant difference of the anti tumor efficacy between STE (3 mg kg^−1^) and the clinical medicine cyclophosphamide (as a positive control group). Additionally, in the pretreatment model, STE (3 g kg^−1^) exhibited excellent anti-tumor effect on malignant proliferation of tumors (Fig. S4†). Taken together, this study further demonstrated that STE acted as an effective agent to guard against tumor *in vivo*. Next, the haematological analysis was performed to evaluate the systemic cytotoxicity of STE *in vivo*.

**Fig. 13:**
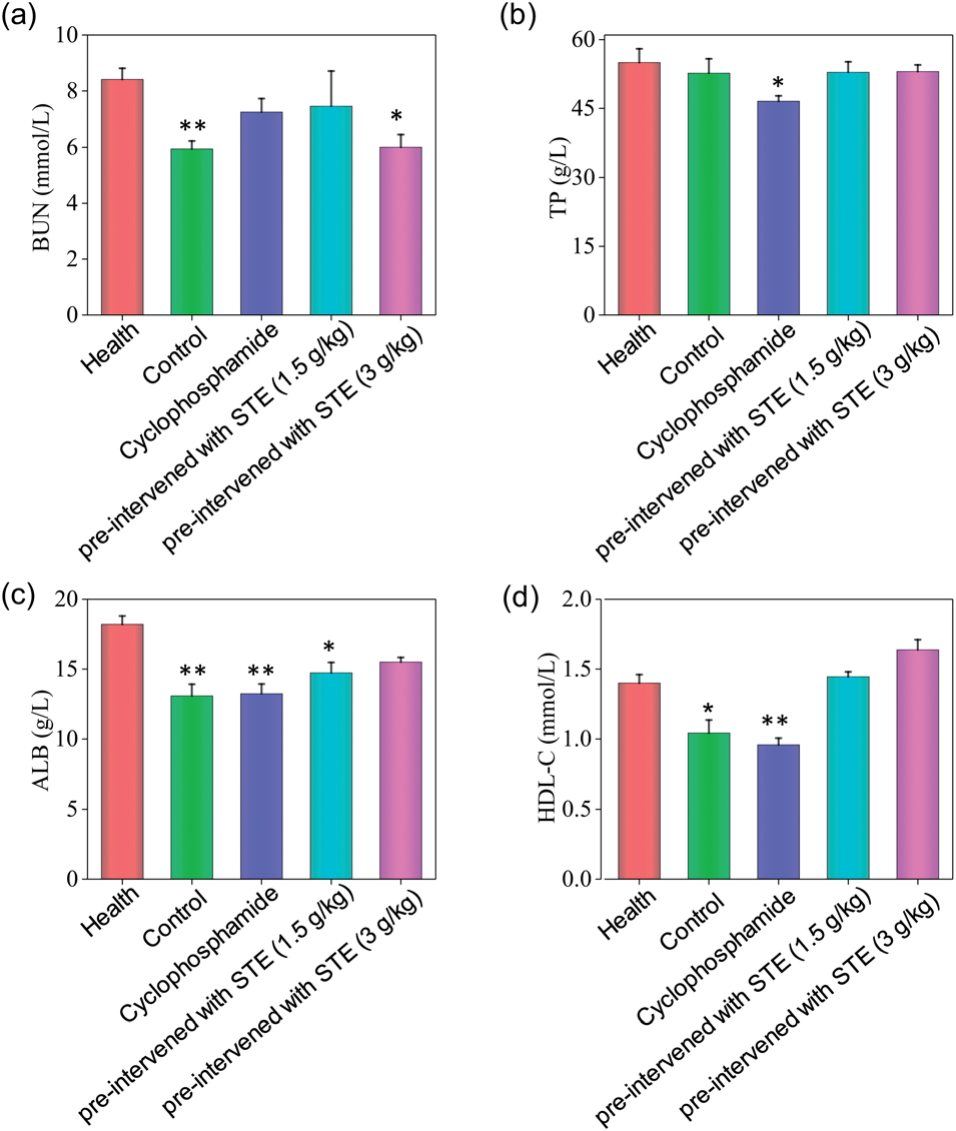
Hematology analysis of nude mice treated with STE. BUN represents blood urea nitrogen, TP represents total protein, ALB represents serum albumin, HDL-C represents high density lipoprotein cholesterol. Value represents means ± SD of triplicates. Significant difference between treatment and the healthy groups is indicated at *P* < 0.05 (*) and *P* < 0.01 (**) level.

As shown in Fig. 13, the hematological analysis data revealed that the mice pre-treated with STE showed no significant dysfunction to liver, kidney, or blood lipid, as reflected by the biochemical indexes of total protein (TP), albumin (ALB), high-density lipoprotein (HDL-C), urea nitrogen (BUN). For instance, the HDL-C index of mice treated with STE (1.5 and 3 g kg^−1^) was 1.49 and 1.66 mmol L^−1^, which was close to that of the healthy group. While the HDL-C index of negative control group and cyclophosphamide group were much lower than healthy group, showing that STE could keep the blood lipid of mice in normal range. The results discussed above showed that the liver and kidney of the tumor-bearing mice and positive control group were damaged, but the STE-treated mice tend to be normal, it was manifested that STE could greatly improve the immunity function of mice and improve their healthy conditions. Taken together, STE brought into play antioxidative and anti-tumor activity, without any obvious concomitant toxicity or side effects around.

## Conclusions

In summary, our work demonstrated that STE and ATE revealed excellent antioxidative activity, which effectively inhibited the ABTS˙^+^ and DPPH˙ free radicals. Additionally, the mechanism of cell death induced by STE and ATE further demonstrated that both oolong tea extracts suppressed the growth of cancer cells by inhibiting ROS generation, subsequently resulting in mitochondrial fragmentation, finally causing cell apoptosis. Taken together, this study demonstrates that Jin Guanyin tea could act as a healthy and prospective substitute for natural antioxidants and a promising prophylactic agent against cancers. This finding provides a great promising nutritional approach to treat diseases related with oxidative stress.

## Conflicts of interest

The authors declare no competing financial interest.

## Abbreviations

STE: Summer tea aqueous extract
ATE: Autumn tea aqueous extract
HPLC: High-performance liquid chromatography
Trolox: 6-Hydroxy-2,5,7,8-tetramethylchromane-2-carboxylic acid
ABTS: 2,2′-Azinobis-3-ethylbenzothiazolin-6-sulfonic acid
DPPH: 1,1-Diphenyl-2-picryhydrazyl
C: (+)-Catechin
CG: Catechin gallate
EC: (−)-Epicatechin
ECG: Epicatechin-3-gallate
EGC: Epigallocatechin
EGCG: Epigallocatechin-3-gallate
CA: Caffeine
A: Adenine
TL: Theophylline
GC: (−)-Gall catechin
GA: Gallic acid
TF1: Theaflavin-3-gallate
TF2: Theaflavin-3′-gallate
TF3: Theaflavin-3,3′-gallate
TF4: Theaflavins
MTT: Thiazolyl blue tetrazolium bromide
PI: Propidium iodide
DAPI: 4′,6-Diamidino-2-phenyindole
DHE: Dihydroethidium
DMEM: Dulbecco's modified Eagle's medium
ROS: Reactive oxygen species

## Supplementary Material

RA-008-C8RA00151K-s001
