# Supplementary material for: Tea regimen, a comprehensive assessment of antioxidant and antitumor activities of tea extract produced by Tie Guanyin hybridization
Source: RSC Adv. 2018 Mar 21;8(21):11305–15. doi: 10.1039/c8ra00151k (PMC9079151; doi:10.1039/c8ra00151k)
Supplement: RA-008-C8RA00151K-s001 [file RA-008-C8RA00151K-s001.pdf]

Electronic Supplementary Material (ESI) for RSC Advances.
This journal is © The Royal Society of Chemistry 2018

Supplementary Information

Tea Regimen, a Comprehensive Assessment of Antioxidant and Antitumor Activities of Tea Extract Produced by Tie Guanyin Hybridization

Xiaobin Zhang[#], Chengli Dai[#], Yuanyuan You, Lizhen He*, Tianfeng Chen*

The First Affiliated Hospital, and Department of Chemistry, Jinan University, Guangzhou 510632, China.

# these authors contributed equally to this study.

E-mail addresses: hlz6371@jnu.edu.cn (L He). tchentf@jnu.edu.cn (T Chen)

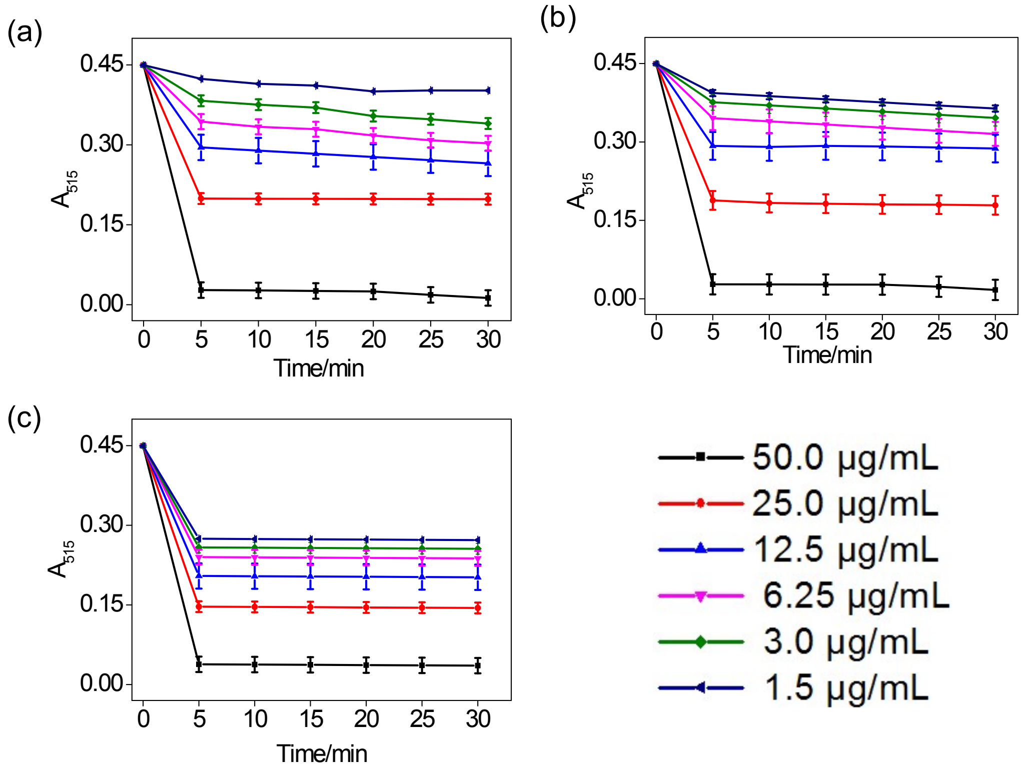

Figure S1 The Absorbance Inhibition level of DPPH• (A_{515}) after addition of Jin Guanyin extracts. (a) Changes in absorbance of DPPH• solution with the addition of STE. (b) Changes in absorbance of DPPH• solution with the addition of ATE. (c) Changes in absorbance of DPPH• solution with the addition of Trolox (standard oxidant). Each value represents means ± SD (n=3).

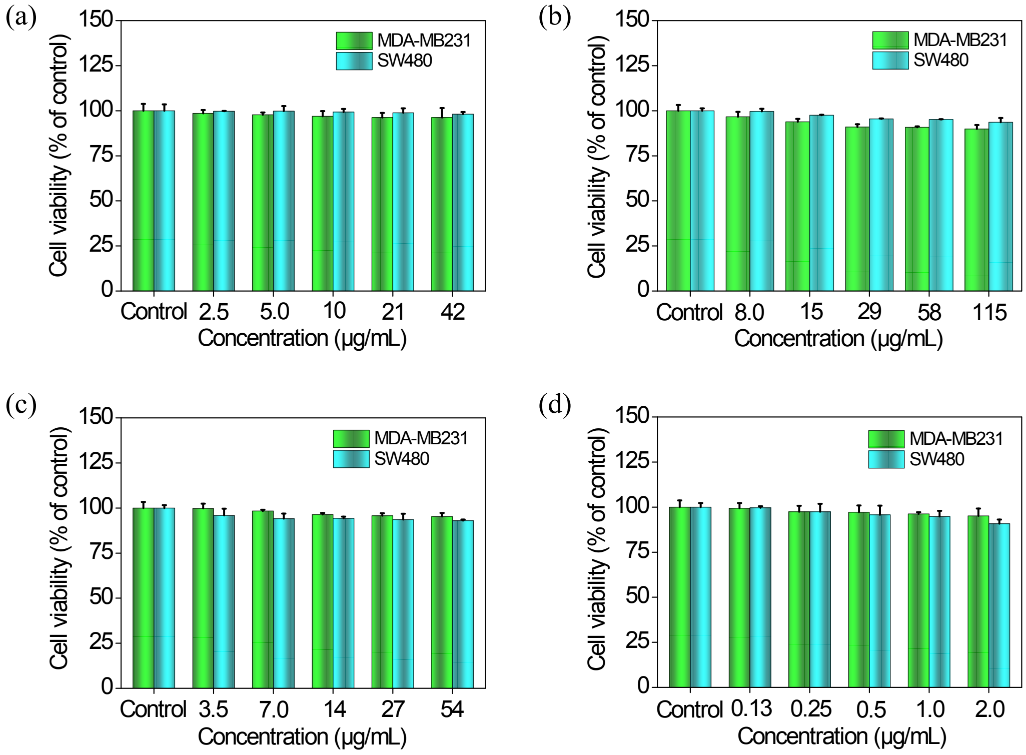

Figure S2 Cytotoxic Effects of the Tea Additive by MTT assay. (a) Cytotoxicity effects of coniferous cherry powder on SW480 (Colon Cancer Cells) and MDA-MB-231 (Breast Cancer Cells). (b) Cytotoxicity effects of sorbitol on SW480 (Colon Cancer Cells) and MDA-MB-231 (Breast Cancer Cells). (c) Cytotoxicity effects of D-mannose on SW480 (Colon Cancer Cells) and MDA-MB-231 (Breast Cancer Cells). (d) Cytotoxicity effects of stearate magnesium stearate on SW480 (Colon Cancer Cells) and MDA-MB-231 (Breast Cancer Cells). Each value represents means ± SD (n = 3).

Table S1. Cytotoxicity Effects of STE and ATE

Tea	IC_{50} (μg/mL^{-1})					
Sample	MDA-MB231	SW480	HepG2	Hela	L02	WI 38
STE	65.90±1.40	109.1±0.22	201.3±6.1	60.90±1.4	171.4±8.7	248±18
ATE	81.40±0.037	50.83±2.1	225.7±0.62	252.0±3.8	199.1±12	71.95±1.9

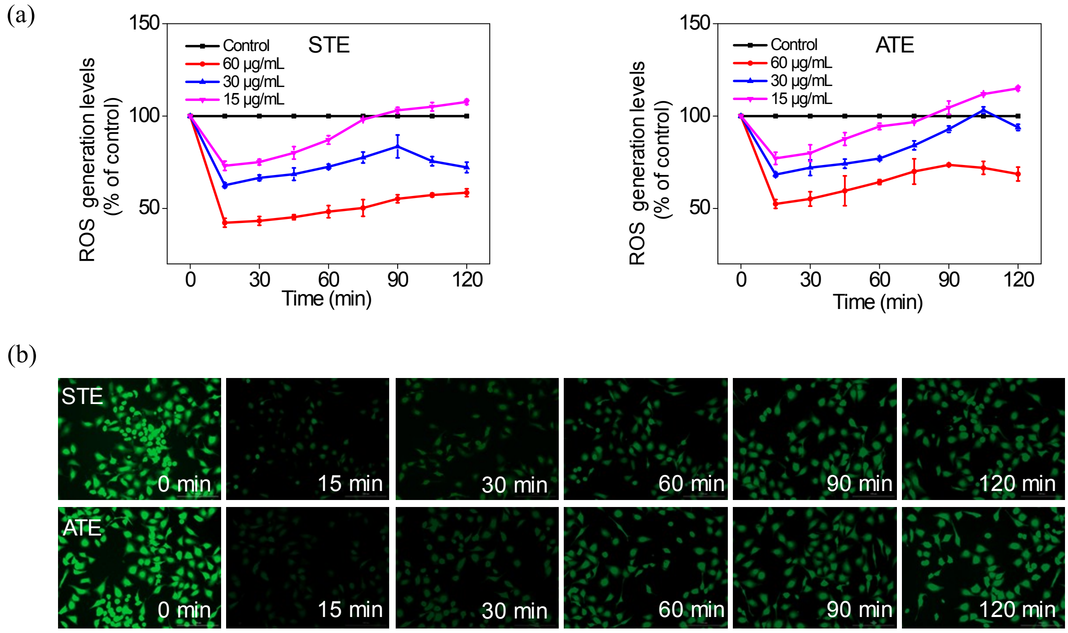

Figure S3 Down-regulations of intracellular ROS levels induced by Jin Guanyin extracts. (a) Intracellular ROS generation after treatment with STE and ATE, respectively. (b) Fluorescence images of ROS generation in MDA-MB-231 cells incubated with STE and ATE at indicated time points. Original magnification: 10×. Each value represents means ± SD (n=3).

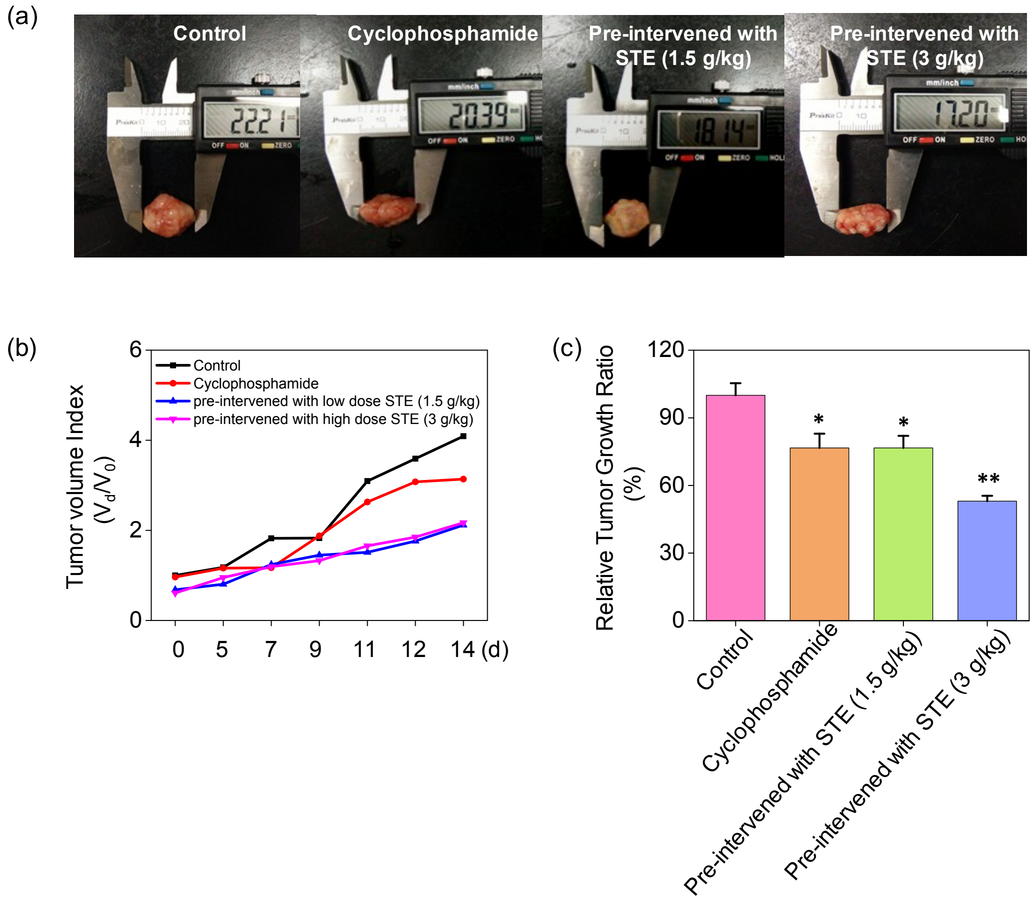

Figure S4 *In vivo* therapeutic effects of STE in MDA-MB-231-bearing nude mice. (a) The tumor photos for each experiment group. (b) The tumor volume index of nude mice through different treatments in 14 days. (c) Relative tumor growth ratio of each group at Day 14. Each value represents means ± SD (n=3). Bars with different characterisitcs are statistically at the $*P < 0.05$ level vs. control, $**P < 0.05$ level vs. control.
